# Hybrid nanofluid flow within the conical gap between the cone and the surface of a rotating disk

**DOI:** 10.1038/s41598-020-80750-y

**Published:** 2021-01-13

**Authors:** Taza Gul, Kashifullah, M. Bilal, Wajdi Alghamdi, M. Imran Asjad, Thabet Abdeljawad

**Affiliations:** 1 Mathematics Department, City University of Science and Information Technology, Peshawar, 25000 Pakistan; 2 Department of Information Technology, Faculty of Computing and Information Technology, King Abdulaziz University, Jeddah, 80261 Saudi Arabia; 3 Department of Mathematics, University of Management and Technology, Lahore, Pakistan; 4 Department of Mathematics and General Sciences, Prince Sultan University, P. O. Box 66833, Riyadh, 11586 Saudi Arabia; 5 Department of Medical Research, China Medical University, Taichung, 40402 Taiwan; 6 Department of Computer Science and Information Engineering, Asia University, Taichung, Taiwan

**Keywords:** Mathematics and computing, Nanoscience and technology

## Abstract

The thermal management of the flow of the hybrid nanofluid within the conical gap between a cone and a disk is analyzed. Four different cases of flow are examined, including (1) stationary cone rotating disk (2) rotating cone stationary disk (3) rotating cone and disk in the same direction and (4) rotating cone and disk in the opposite directions. The magnetic field of strength $B_{0}$ is added to the modeled problem that is applied along the z-direction. This work actually explores the role of the heat transfer, which performs in a plate-cone viscometer. A special type of hybrid nanoliquid containing copper *Cu* and magnetic ferrite *Fe*_3_*O*_4_ nanoparticles are considered. The similarity transformations have been used to alter the modeled from partial differential equations (PDEs) to the ordinary differential equations (ODEs). The modeled problem is analytically treated with the Homotopy analysis method HAM and the numerical ND-solve method has been used for the comparison. The numerical outputs for the temperature gradient are tabulated against physical pertinent variables. In particular, it is concluded that increment in volume fraction of both nanoparticles $\left( {\phi_{{Fe_{3} O_{4} }} ,\phi_{Cu} } \right)$ effectively enhanced the thermal transmission rate and velocity of base fluid. The desired cooling of disk-cone instruments can be gained for a rotating disk with a fixed cone, while the surface temperature remains constant.

## Introduction

The study exhibits that the cone-disk devices have several practical and technical applications, like in the stability analysis of an Oldroyd-B fluid creeping flow^1^, medical purposes^2^, in the calculation of viscosity of fluid using viscosimetry^3^, for gas turbines in a conical diffuser in the cooling system to compress air^4^. Choi^5^ is the pioneer to use the nano sized small particles of the metals, carbides and oxides in the base fluids to enhance the thermal conductivity of the base fluids. The present work also deals with the heat transmission through hybrid nanoliquid passing between the gap of a disk and cone, in which either both are rotating in the same direction or in different with angular velocity, or maybe one remains stationary with respect to another. Such type of studied attract a number of researchers to examine its behavior. Turkilmazoglu^6^ used semi- analytical method (HAM) and investigated streamline flow on a spinning cone, and produced well documented outputs successfully. Chamkha et al.^7^ numerically computed the time dependent problem with heat and mass transfer from a vertical spinning cone. A series of stability analysis of boundary layer was later studied by Garrett et al.^8,9^, plunging into the convective or absolute behavior of instability due to the revolving cone. The magneto hydrodynamics MHD nanoliquid flow over a spinning cone with the thermophoresis and Brownian motion influence was illustrated in^10^. Similarity solutions of the compressible laminar flows subject to surface mass flux over a group of revolving cones were scrutinized in^11^.

With the immediate development of nanotechnology and modern sciences. The nanomaterials has gained tremendous attention from many of the researchers. The small particles in the nanometer sized are stabile dispersed in the base fluids to perform nanofluids. The metal oxides, carbon materials and so on are used as the nanoparticles. Nanofluids, are mainly used in the thermal engineering, fiber technology and electronic devices. The heat and mass transfer with nanoliquid flow runs over revolving disk has been a great area of research, because of its vast applications in the field of electronic devices and heat exchanger^12^. Rasool and Zhang^13^, discussed the effect of Darcy Forchheimer visco-elastic nanofluid flow bounded by a non-linear stretching sheet/surface with Cattaneo-Christov heat—mass flux. Shirejini et al.^14^ have used the nanofluid to restore the drop through heat transfer rate utilizing the gyrating scheme. The thermal transmission of an electrically conducting fluid over a spinning infinite disk is scrutinized by^15^. The areas of applications of such type problems are in computer devices for storage purpose, rotating machinery, thermal energy generating system, electronic instruments, geothermal industry, gas turbines, chemical processes, rotating machinery, various types of medical apparatus etc. Turkyilmazoglu^16^ examined fluid flow and heat transmission on a vertically moving spinning disk. The heat transfers and induced flow in a quiescent fluid due to a spinning cone^17^. Kumar et al.^18^ used finite element technique and noted the entropy generation of radiative flow of nanoliquid consist of copper and aluminum oxide nano size particles between the spaces of two coaxial revolving disks. Bhattacharyya et al.^19^ simulated the heat flux on the flow of CNTs carbon nanotubes nanoliquid amongst the two coaxial stretchable spinning disks. The fluid flow over a revolving disk is recently examined by Hafeez et al.^20^. The numerical study of the non-Newtonian water/Al_2_O_3_ nanoliquid with 0–4% nano size particles inside a two-dimensional square cavity with cold and hot lid-driven motion is simulated at Richardson numbers using Fortran code^21^. The Maxwell fluid flow with the effect of homogeneous–heterogeneous reactions between two revolving disks and heat conduction was scrutinized by Ahmed et al.^22^. Also, a numerical study has been fulfilled to explore the fluid and thermal behaviors in different shaped enclosures filled with an Al_2_O_3_/water nanofluid and with a rectangular hot obstacle was investigated by Rashidi et al.^23–25^.

The basic principle of MHD is to regulate fluid flow. For the appropriate cooling purpose, the higher thermal conductivity and better heat transmission rate are only possible, if the phenomena would be considered under the magnetic force. The malignant tumor, arthritis, blood pressure, and brain therapy are the known uses of magnetic effect. Siddiqui et al.^26^ investigated the MHD movement of fluid flow with the application of respiratory track to monitor diseases. The Keller-box method is used in^27^ to solve numerically a problem of gyrating MHD flow over a porous surface. Subhani and Nadeem^28^ examined the time-dependent MHD flow of hybrid nanoliquid over the rotating porous surface, by considering fluid theory. Lokesh et al.^29^ highlighted numerically the chemical reaction of the 3D Casson nanofluid flow over an expanding surface. An unsteady three-dimensional MHD flow of nanoliquid is investigated by Rauf et al.^30^, as a result of rotation of infinite disc with periodic oscillation dependent on time. Oyelakin et al.^31^ revealed the impact of the velocity slip in a tangent hyperbolic nanoliquid flow and heat transmission features. The key focus of the work in^32^ is to inspect the flow characters of Cu-*Al*_2_*O*_3_/water hybrid nanofluid with the mutual impact of joule heating, suction and MHD over an extending/shrinking surface. Tlili et al.^33^ scrutinized a magnetic flow of hybrid nanoliquid through a stretched plane with slip impacts. In this modern era of science and technology, the hybrid nanoliquid has gained a great attention among the researchers, due to its potential and remarkable thermal characters, which delivered better outputs as compared to simple nanoliquid in improving the heat transmission rate. On the basis of experimental studies, it is concluded that from 5 to 55% volume fraction of nano material (1–100 nm) are considered for a high thermal conductivity and better thermal transmission rate of carrier fluids^34^. An experimental survey on the influence of concentration and temperature of tiny particles on the viscous property of ZnO–MWCNTs/engine oil hybrid nano lubricant is examined by^35^. The one phase nanoliquids hydrodynamic stability based on the linear stability concept is highlighted by^36^. Bovand et al.^37^ simulated the laminar flow of aluminum-oxide Al_2_O_3_-water nanoliquids between the gaps of two parallel plates at fixed temperatures. The influence of temperature differences between the walls and fluid, including the thermophoresis force was studied in the model. The MHD has a positive upshot on the reduction of the unsteady forces, and a diverse impact on the thermal property. The addition of tiny particles to the base fluid is a well-known technique for thermal-characteristics improvement, that lead to enhancement in drag forces^38^. Additionally, some other types of nanoparticles used in hybrid nanofluid recently studied by Waini et al.^39–42^ for the enhancement of heat transfer. It is also discovered that 5% volume fraction of nanoparticles in base fluid is more effective for the maximum heat transfer rate. The water based *Fe*_*3*_*O*_*4*_ nanoliquid, when its volume fraction is 12–15% shows positive effects on Nusselt number^41^. Yahaya et al.^43^ studied heat transmission through Cu-*Al*_2_*O*_3_/H_2_*O* hybrid nanoliquid past over a stretching sheet. The multiple solution of Cu-*Al*_2_*O*_3_/H_2_*O* hybrid nanoliquid consist of nanoparticles over shrinking surface was scrutinized by^44^. The unique solution exists when $\lambda_{c} > - 1$ ($\lambda_{c}$ is the plate velocity), and duality exists when it is in the range of $\lambda_{c} < - 1$. As the magnetic effect enhances the solution duality range increase^45^. Lund et al.^46^ found the dual solution for MHD flow Cu-*Fe*_3_*O*_4_/H_2_*O* hybrid nanoliquid under the influence of viscous dissipation.

The above studies, witness that, no effort so far has been made to scrutinize the three-dimensional hybrid nanoliquid flow model about the disk and cone as moving or stationary, under the influence of magnetic field. This work actually explores the role of copper Cu and magnetic ferrite *Fe*_3_*O*_4_ nanoparticles on the thermophysical properties of water. Which has several important applications in sciences and technology. The second priority is to extend the idea of Refs.^47,48^, which also consist most relevant studies related to the present model. To develop a mathematical model for rotating disk and cone, which are considered as moving or stationary, in both case counter rotating or co-rotating. The flow equations are diminished to ordinary system, and then tackled with HAM. Influences of physical pertinent variables on velocity and temperature are highlighted through Figures. The numerical outputs for surface drag force and temperature gradient are tabulated against interesting physical entities. The originality of the current work is pointed out as.

1. In the present work the three dimensional *Cu* + *Fe*_3_*O*_4_/*H*_2_*O* hybrid nanofluid flow is considered while the existing study^47,48^ is limited to the viscous fluid and nanofluids including CNTs.
2. The magnetic field is imposed vertically to the flow pattern in the present work while the existing work^47^ is without the magnetic field.
3. Four different cases for the flow between a disk and cone (1) stationary disk rotating cone (2) rotating disk stationary cone (3) counter rotating of the disk and cone (4) co-rotating of the disk and cone are examined and discussed and this idea extended to both the velocity and temperature profiles.
4. The HAM technique and BVPh 2.0 package have been used for the solution of the nonlinear problem and this method is compared with the numerical (ND-solve) method.
5. It has been observed that hybrid nanofluids improve the thermal efficiency of the base fluids rapidly as compared to the other fluids.

## Mathematical formulation

**Figure 1:**
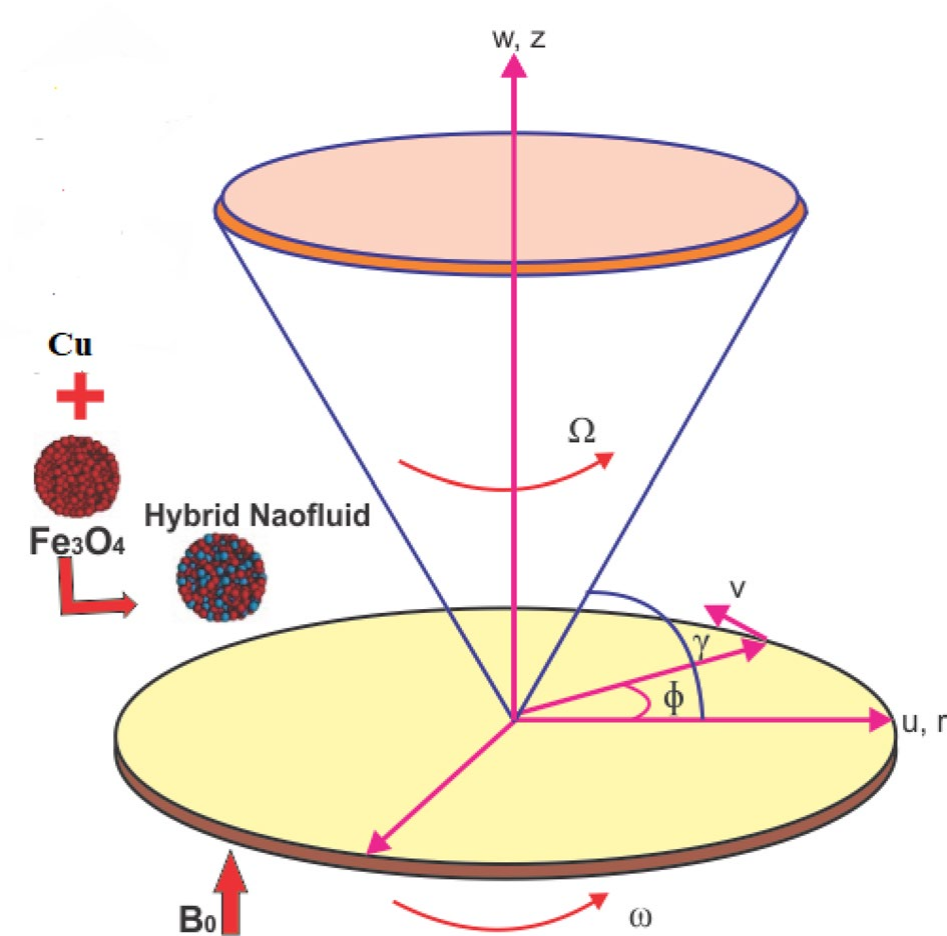
Geometry of the problem.

Consider a disk and cone with an incompressible hybrid nanoliquid under the influence of magnetic field is under consideration. Both tools (disk and cone) are assumed to be either rotating or stationary with angular velocity in the cylindrical coordinate $\left( {r,\varphi ,z} \right)$. The $\omega$ and $\Omega$ highlight the disk and cone angular velocities respectively. *B*_0_ is the strength of magnetic field that is applied along z-direction, whereas the induced magnetic field is neglected. The flow mechanism is illustrated in Fig. 1. Heat transportation modeling is computed with the addition of viscous dissipation. The phenomenon is successfully applied on the surface of the disk with a radially variable wall temperature $T_{w} = T_{\infty } + cr^{n}$, here *n* and *c* are kept fixed, where $T_{\infty }$ is the cone wall^47^. Within the conical gap, *p* is the pressure depending on both axial *z* and radial *r* distances. The governing equations on the basis of above assumption can be stated as^47,48^:

1 $$\frac{\partial u}{{\partial r}} + \frac{\partial w}{{\partial z}} + \frac{u}{r} = 0,$$

2 $$\rho_{hnf} \left[ {u\frac{\partial u}{{\partial r}} + w\frac{\partial u}{{\partial z}} - \frac{{v^{2} }}{r}} \right] = - \frac{\partial p}{{\partial r}} + \mu_{hnf} \left[ {\frac{{\partial^{2} u}}{{\partial r^{2} }} + \frac{{\partial^{2} u}}{{\partial z^{2} }} + \frac{1}{r}\frac{\partial u}{{\partial r}} - \frac{u}{{r^{2} }}} \right] - \sigma_{hnf} B_{0}^{2} u,$$

3 $$\rho_{hnf} \left[ {u\frac{\partial v}{{\partial r}} + w\frac{\partial v}{{\partial z}} + \frac{uv}{r}} \right] = \mu_{hnf} \left[ {\frac{{\partial^{2} v}}{{\partial r^{2} }} + \frac{{\partial^{2} v}}{{\partial z^{2} }} + \frac{1}{r}\frac{\partial v}{{\partial r}} - \frac{v}{{r^{2} }}} \right] - \sigma_{hnf} B_{0}^{2} v,$$

4 $$\rho_{hnf} \left[ {u\frac{\partial w}{{\partial r}} + w\frac{\partial w}{{\partial z}}} \right] = - \frac{\partial p}{{\partial z}} + \mu_{hnf} \left[ {\frac{{\partial^{2} w}}{{\partial r^{2} }} + \frac{{\partial^{2} w}}{{\partial z^{2} }} + \frac{1}{r}\frac{\partial w}{{\partial r}}} \right],$$

5 $$\left( {\rho cp} \right)_{hnf} \left[ {u\frac{\partial T}{{\partial r}} + w\frac{\partial T}{{\partial z}}} \right] = k_{hnf} \frac{{\partial^{2} T}}{{\partial z^{2} }} + \sigma_{hnf} B_{0}^{2} \left( {u^{2} + v^{2} } \right),$$

where $\left( {u,v,w} \right)$ are the velocity components along $\left( {r,\varphi ,z} \right)$ directions, $B_{0}$ is the magnetic strength, *p* is the fluid pressure. While $k_{hnf}$, $\rho_{hnf}$, $\nu_{hnf}$, $\mu_{hnf}$, $\left( {\rho c_{p} } \right)_{hnf}$ and $\sigma_{hnf}$ is the thermal conductivity, density, dynamic viscosity, heat capacitance and electrical conductivity of hybrid nanoliquid respectively.

### Boundary conditions

The obligatory boundary conditions are as

6 $$\begin{aligned} & u = 0, \, v = \omega \,r,T = T_{w} \,, \, w = 0, \, \,\,\,{\text{z = 0}} \\ & u = 0, \, v = \Omega \,r, \, T = T_{\infty } ,\,w = 0,\,\,z = r\tan \gamma \\ \end{aligned}$$

Here $\gamma$ specified the gap angle between the cone and disk.

### Similarity conversion

In order to nondimensionalization, we adopt the following similarity transformation^47^:

7 $$\begin{aligned} & u = \frac{{\upsilon_{f} F(\eta )}}{r}{\text{ = U}}_{w} F\left( \eta \right){,}\,\,v = \frac{{\upsilon_{f} G(\eta )}}{r} = {\text{U}}_{w} G\left( \eta \right),\,\,w = \frac{{\upsilon_{f} H(\eta )}}{r}{\text{U}}_{w} H\left( \eta \right),{\text{ p = }}\frac{{\rho \upsilon_{f}^{2} P}}{{r^{2} }}{\text{U}}_{w}^{2} \rho \cdot p, \\ & \eta = \frac{z}{r},\,\,\Theta = \frac{{T - T_{\infty } }}{{T_{w} - T_{\infty } }},\,M = \frac{{\nu_{f} \sigma_{f} \,B_{0}^{2} }}{{\rho_{f} U_{w}^{2} }}\,,\,\,\Pr = \frac{{\mu_{f} Cp}}{{k_{f} }}. \\ \end{aligned}$$

Here Uw is used as the surface velocity, Pr is the Prandtl number and M is the magnetic field. Now, with the help of these transformations as in Eq. (7), the modeled Eqs. (2–5) and their boundary conditions modify to the following fashion:

8 $$H^{\prime } - \eta F^{\prime } ,$$

9 $$\begin{aligned} & (1 + \eta^{2} )F^{\prime \prime } + 3\eta F^{\prime } + \left( {1 - \phi_{{Fe_{3} O_{4} }} } \right)^{2.5} \left( {1 - \phi_{Cu} } \right)^{2.5} \left[ {\left( {1 - \phi_{Cu} } \right)\left( {1 - \left( {1 - \frac{{\rho_{{Fe_{3} O_{4} }} }}{{\rho_{f} }}} \right)\phi_{{Fe_{3} O_{4} }} } \right) + \phi_{Cu} \left( {\frac{{\rho_{Cu} }}{{\rho_{f} }}} \right)} \right]\left[ {\eta FF^{\prime } - HF^{\prime } + F^{2} - G^{2} } \right] \\ & \quad + \left( {1 - \phi_{{Fe_{3} O_{4} }} } \right)^{2.5} \left( {1 - \phi_{Cu} } \right)^{2.5} \left[ {2p + \eta p^{\prime } - MF} \right] = 0, \\ \end{aligned}$$

10 $$\begin{aligned} & (1 + \eta^{2} )G^{{\prime {\prime }}} + + 3\eta G^{\prime } - \left( {1 - \phi_{{Fe_{3} O_{4} }} } \right)^{2.5} \left( {1 - \phi_{Cu} } \right)^{2.5} \left[ {\left( {1 - \phi_{Cu} } \right)\left( {1 - \left( {1 - \frac{{\rho_{{Fe_{3} O_{4} }} }}{{\rho_{f} }}} \right)\phi_{{Fe_{3} O_{4} }} } \right) + \phi_{Cu} \left( {\frac{{\rho_{Cu} }}{{\rho_{f} }}} \right)} \right]\left[ {\eta FG^{\prime } - HG^{\prime } } \right] \\ & \quad - \left( {1 - \phi_{{Fe_{3} O_{4} }} } \right)^{2.5} \left( {1 - \phi_{Cu} } \right)^{2.5} MG = 0, \\ \end{aligned}$$

11 $$\begin{aligned} & (1 + \eta^{2} )H^{{\prime {\prime }}} + + 3\eta H^{\prime } + \left( {1 - \phi_{{Fe_{3} O_{4} }} } \right)^{2.5} \left( {1 - \phi_{Cu} } \right)^{2.5} \left[ {\left( {1 - \phi_{Cu} } \right)\left( {1 - \left( {1 - \frac{{\rho_{{Fe_{3} O_{4} }} }}{{\rho_{f} }}} \right)\phi_{{Fe_{3} O_{4} }} } \right) + \phi_{Cu} \left( {\frac{{\rho_{Cu} }}{{\rho_{f} }}} \right)} \right]\left[ {\eta FH^{\prime } - HH^{\prime } + H + FH} \right] \\ & \quad - \left( {1 - \phi_{{Fe_{3} O_{4} }} } \right)^{2.5} \left( {1 - \phi_{Cu} } \right)^{2.5} p^{\prime} = 0, \\ \end{aligned}$$

12 $$\begin{aligned} & \frac{{k_{hnf} }}{{k_{nf} }}\left[ {(1 + \eta^{2} )\Theta^{\prime \prime } + + \eta (1 - 2n)\Theta^{\prime } + n^{2} \Theta } \right] + \Pr \left[ {(1 - \phi_{Cu} )\left( {1 - \left( {1 - \frac{{(\rho C_{p} )_{{Fe_{3} O_{4} }} }}{{(C_{p} \rho )_{f} }}\phi_{{Fe_{3} O_{4} }} } \right) + \frac{{(\rho C_{p} )_{Cu} }}{{(C_{p} \rho )_{f} }}\phi_{Cu} } \right)} \right]\left[ {\eta F\Theta^{\prime } - nF\Theta - H\Theta^{\prime } } \right] \\ & \quad + \frac{M}{{\left( {1 - \phi_{Cu} } \right)^{2.5} \left( {1 - \phi_{{Fe_{3} O_{4} }} } \right)^{2.5} }}\left( {F^{2} + G^{2} } \right) = 0, \\ \end{aligned}$$

The modified conditions are:

13 $$\begin{aligned} & F(0) = H(0) = 0,\,\,G(0) = {\text{Re}}_{\omega } ,\Theta (0) = 1, \\ & F(\eta_{0} ) = H(\eta_{0} ) = 0,G(\eta_{0} ) = {\text{Re}}_{\Omega } ,\Theta (\eta_{0} ) = 0. \\ \end{aligned}$$

The volumetric fraction of *Fe*_3_*O*_4_ and *Cu* are demonstrated through $\phi_{{Fe_{3} O_{4} }}$ and $\phi_{Cu}$. While $k_{hnf}$, $k_{f}$ is the thermal conductivity of hybrid nanoliquid and water.

### Thermo-physical properties

The different thermal characteristics of hybrid nanoliquid and water as follows^49^:

14 $$\begin{aligned} & \upsilon_{hnf} = \frac{{\mu_{hnf} }}{{\rho_{hnf} }},\mu_{hnf} = \frac{{\mu_{f} }}{{(1 - \phi_{{Fe_{3} O_{4} }} )^{5/2} (1 - \phi_{Cu} )^{5/2} }},\frac{{(\rho )_{hnf} }}{{(\rho )_{f} }} = \left( {1 - \phi_{Cu} } \right)\left( {1 - \left( {1 - \frac{{\rho_{{Fe_{3} O_{4} }} }}{{\rho_{f} }}} \right)\phi_{{Fe_{3} O_{4} }} } \right) + \phi_{Cu} \left( {\frac{{\rho_{Cu} }}{{\rho_{f} }}} \right), \\ & \frac{{(\rho C_{p} )_{hnf} }}{{(\rho C_{p} )_{f} }} = (1 - \phi_{Cu} )\left\{ {1 - \left( {1 - \frac{{(\rho C_{p} )_{{Fe_{3} O_{4} }} }}{{(\rho C_{p} )_{f} }}} \right)\phi_{{Fe_{3} O_{4} }} } \right\} + \frac{{(\rho C_{p} )_{Cu} }}{{(\rho C_{p} )_{f} }}\phi_{Cu} , \\ & \frac{{\sigma_{hnf} }}{{\sigma_{bf} }} = \left[ {\frac{{\left( {\sigma_{Cu} - \sigma_{bf} } \right)3\phi_{Cu} }}{{\left( {\sigma_{Cu} + 2\sigma_{bf} } \right) + \left( {\sigma_{bf} - \sigma_{Cu} } \right)\phi_{Cu} }} + 1} \right],\frac{{\sigma_{bf} }}{{\sigma_{f} }} = \left[ {\frac{{\left( {\sigma_{f} - \sigma_{{Fe_{3} O_{4} }} } \right)3\phi_{{Fe_{3} O_{4} }} }}{{\left( {\sigma_{{Fe_{3} O_{4} }} - \sigma_{f} } \right) - \left( {\sigma_{{Fe_{3} O_{4} }} + 2\sigma_{f} } \right)}} + 1} \right], \\ & \frac{{k_{hnf} }}{{k_{nf} }} = \frac{{k_{Cu} + 2k_{nf} - 2\phi_{Cu} (k_{nf} - k_{Cu} )}}{{k_{Cu} + 2k_{nf} + \phi_{Cu} (k_{nf} - k_{Cu} )}},\,\,\frac{{k_{nf} }}{{k_{f} }} = \frac{{k_{{Fe_{3} O_{4} }} + 2k_{f} - 2\phi_{{Fe_{3} O_{4} }} (k_{f} - k_{{Fe_{3} O_{4} }} )}}{{k_{{Fe_{3} O_{4} }} + 2k_{f} + \phi_{{Fe_{3} O_{4} }} (k_{f} - k_{{Fe_{3} O_{4} }} )}}. \\ \end{aligned}$$

Here $\rho_{hnf} ,\upsilon_{hnf} ,Cp_{hnf} ,k_{hnf} ,\sigma_{hnf}$ are the density, kinematic viscosity, specific heat, thermal conductivity and electrical conductivity of the hybrid nanofluids. The dimensionless shape of the heat transmission rate from the disk and cone surfaces are defined as:

15 $$Nu_{d} = - \frac{{k_{hnf} }}{{k_{nf} }}\Theta^{{\prime }} (0),Nu_{c} = - \frac{{k_{hnf} }}{{k_{nf} }}\Theta^{{\prime }} (\eta_{0} ).$$

$Nu_{d}$ is the Nusselt number for the disc and $Nu_{c}$ for the cone.

## Problem solution

Liao^50–52^ for the 1st time introduced HAM method for the solution of nonlinear differential equations. In the present paper, we also tackled the modeled equations through HAM. The HAM method climbed by Liao addresses all highly nonlinear problems with sufficient choice to select parameters values to permit a convergent series solution. Contrary to numerical schemes, HAM can also tackle the far field boundary value problems. Salient characteristics of the said scheme are, HAM solutions are free from the selection of small/large parameters, unlike the perturbation schemes. The convergence of the series solutions is controlled by the auxiliary parameter instead of the physical parameter. HAM also provides us autonomy for the choice of initial guess estimates by keeping in view the physical system of the problem under consideration. This may be of polynomial, exponential, trigonometric or logarithmic nature. Many studies^53,54^ have verified the validity and effectiveness of this method. To reveal the convergence rate, the sum of residual error is calculated through BVP 2.0 package^55,56^. The preliminary approximations are selected in this method which satisfy the initial and boundary conditions. The linear operators are used to find the initial guesses for the model problem. In the HAM technique initial guesses are required to run the Mathematica code. The convergence is totally dependent on the initial guesses (Trial solution).

The initial approximation for velocity $F_{0} ,G_{0} ,H_{0}$, temperature $\Theta_{0}$ are given as

16 $$F_{0} \left( \eta \right) = 0,G_{0} \left( \eta \right) = \frac{{\left( {{\text{Re}}_{\Omega } - {\text{Re}}_{\omega } } \right)}}{{\eta_{0} }}\eta + {\text{Re}}_{\omega } ,H_{0} \left( \eta \right) = 0,\Theta_{0} \left( \eta \right) = \frac{{\eta_{0} - \eta }}{{\eta_{0} }}.$$

The linear operators for the proposed problem are suggested as:

17 $${\mathscr{L}}_{\,F} (F) = F^{{{\prime \prime }}}, {\mathscr{L}}_{\,H} (H) = H^{{{\prime \prime }}}, {\mathscr{L}}_{\,G} (G) = G^{{{\prime \prime }}}, {\mathscr{L}}_{\,\Theta } (\Theta ) = \Theta^{{{\prime \prime }}} .$$

The expand form of

18 $${\mathscr{L}}_{\,F} \left[ {\chi_{1} + \chi_{2} \eta } \right] = 0,{\mathscr{L}}_{\,G} \left[ {\chi_{3} + \chi_{4} \eta } \right] = 0,{\mathscr{L}}_{\,H} \left[ {\chi_{5} + \chi_{6} \eta } \right] = 0,\,{\mathscr{L}}_{\Theta } \left[ {\chi_{7} + \chi_{8} \eta } \right] = 0. \,$$

After applying Liao’s idea (BVPh 2.0 package) to Eqs. (9–12) as:

19 $$\varepsilon_{m}^{F} = \frac{1}{n + 1}\sum\limits_{x = 1}^{n} {\left[ {N_{F} \left( {\sum\limits_{y = 1}^{m} {F(\eta ),\sum\limits_{y = 1}^{m} {G(\eta ),\sum\limits_{y = 1}^{m} {H(\eta )} } } } \right)_{\eta = x\delta \eta } } \right]^{2} } ,$$

20 $$\varepsilon_{m}^{G} = \frac{1}{n + 1}\sum\limits_{x = 1}^{n} {\left[ {N_{G} \left( {\sum\limits_{y = 1}^{m} {F(\eta ),\sum\limits_{y = 1}^{m} {G(\eta ),\sum\limits_{y = 1}^{m} {H(\eta )} } } } \right)_{\eta = x\delta \eta } } \right]^{2} } ,$$

21 $$\varepsilon_{m}^{H} = \frac{1}{n + 1}\sum\limits_{x = 1}^{n} {\left[ {N_{F} \left( {\sum\limits_{y = 1}^{m} {H(\eta )} } \right)_{\eta = x\delta \eta } } \right]^{2} } ,$$

22 $$\varepsilon_{m}^{\Theta } = \frac{1}{n + 1}\sum\limits_{x = 1}^{n} {\left[ {N_{\Theta } \left( {\sum\limits_{y = 1}^{m} {F(\eta ),\sum\limits_{y = 1}^{m} {G(\eta ),\sum\limits_{y = 1}^{m} {\Theta (\eta )} } } } \right)_{\eta = x\delta \eta } } \right]^{2} } ,$$

The total sum and the square residual are stated as

23 $$\varepsilon_{m}^{t} = \varepsilon_{m}^{F} + \varepsilon_{m}^{G} + \varepsilon_{m}^{H} + \varepsilon_{m}^{\Theta } .$$

The total square residual error is used to calculate the convergence of the proposed problem. The BVPh 2.0 package is also used to obtain the range of the physical parameters.

## Result and discussion

**Figure 2:**
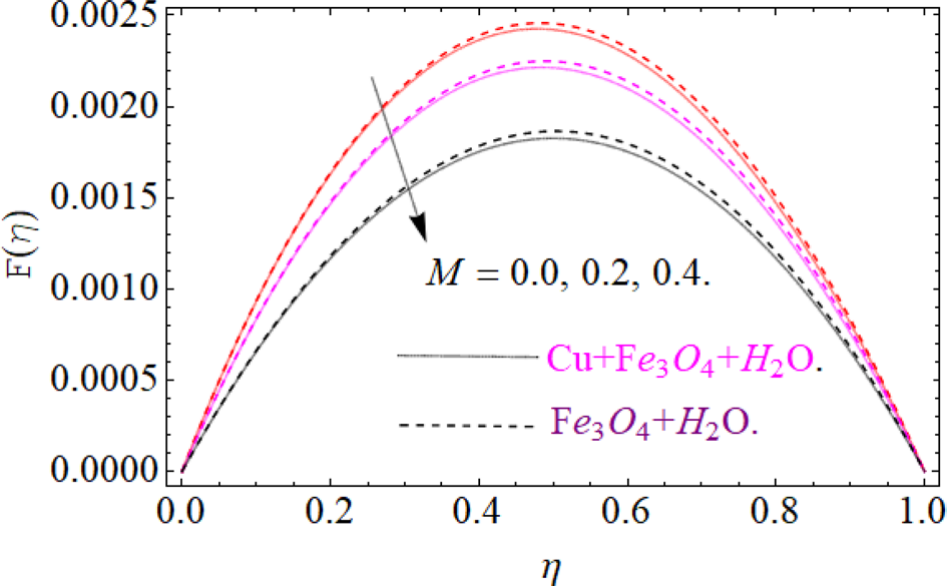
Impression of *M* on F(*η*), When $\phi_{{Fe_{3} O_{4} }} = \phi_{Cu} = 0.02,{\text{Ec}} = 0.1,\Pr = 6.2,{\text{Re}}_{\omega } = 0.3,{\text{Re}}_{\Omega } = 0.13$.

**Figure 3:**
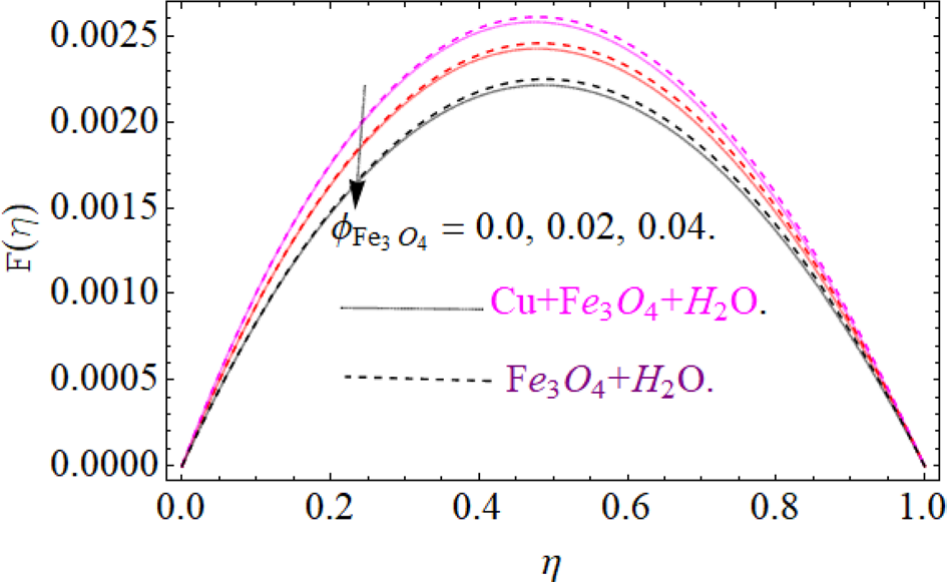
Impression of $\phi_{{Fe_{3} O_{4} }}$ on F(*η*), When $M = 0.2,\phi_{Cu} { = 0}{\text{.02,Ec}} = 0.1,\Pr = 6.2,{\text{Re}}_{\omega } = 0.3,{\text{Re}}_{\Omega } = 0.13$.

**Figure 4:**
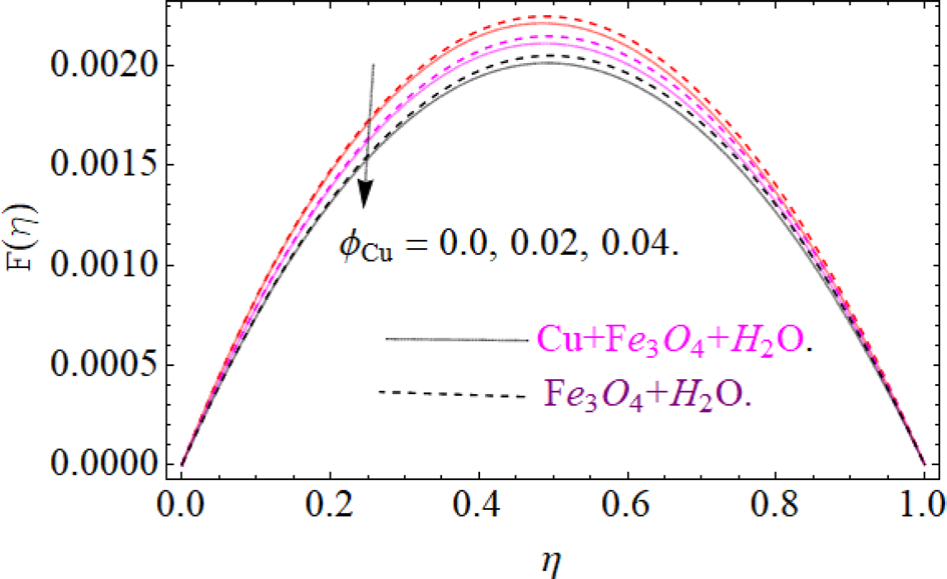
Impression of $\phi_{Cu}$ on F(*η*), When $M = 0.2,\phi_{{Fe_{3} O_{4} }} = 0.02,\,{\text{Ec = 0}}{.1,}\,{\text{Pr = 6}}{.2}$.

The motive behind this section is to investigate the nature of different physical entities of versus velocity and temperature profiles. Figure 1 exhibit the flow mechanism of rotating cone and disk. Features of the parameter *M* (magnetic number) on the axial velocity $F\left( \eta \right)$ profile are illustrated in Fig. 2. It can be noticed that the rising credit of magnetic parameter declines the fluid velocity $F\left( \eta \right)$ of both copper *Cu* and magnetic ferrite $Fe_{3} O_{4}$ hybrid nanoliquid. Physically, the Lorentz force is generated by magnetic number *M,* which retard the fluid velocity, as a result the velocity decreases. Figure 3 and 4 revealed the impact of volume fraction parameters $\left( {\phi_{{Fe_{3} O_{4} }} ,\phi_{Cu} } \right)$ on the axial velocity $F\left( \eta \right)$ profile. The positive increment in $\phi_{{Fe_{3} O_{4} }}$ and $\phi_{Cu}$ enhancing the boundary layer thickness, which decline the velocity profile.

**Figure 5:**
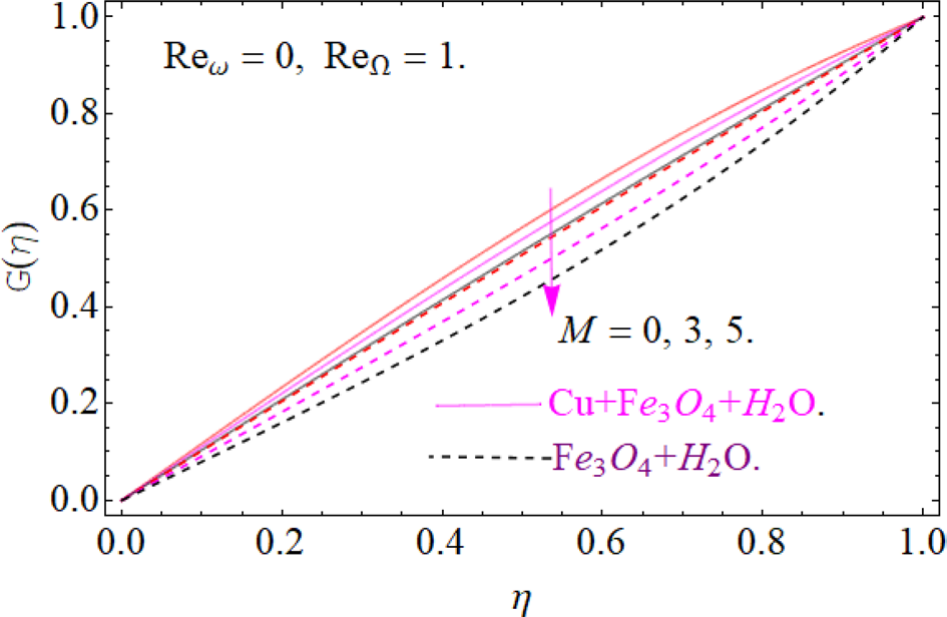
Impression of *M* on G(*η*), When $\phi_{{Fe_{3} O_{4} }} = \phi_{Cu} = 0.02,\,{\text{Ec = 0}}{.1,}\,{\text{Pr = 6}}{.2}$.

**Figure 6:**
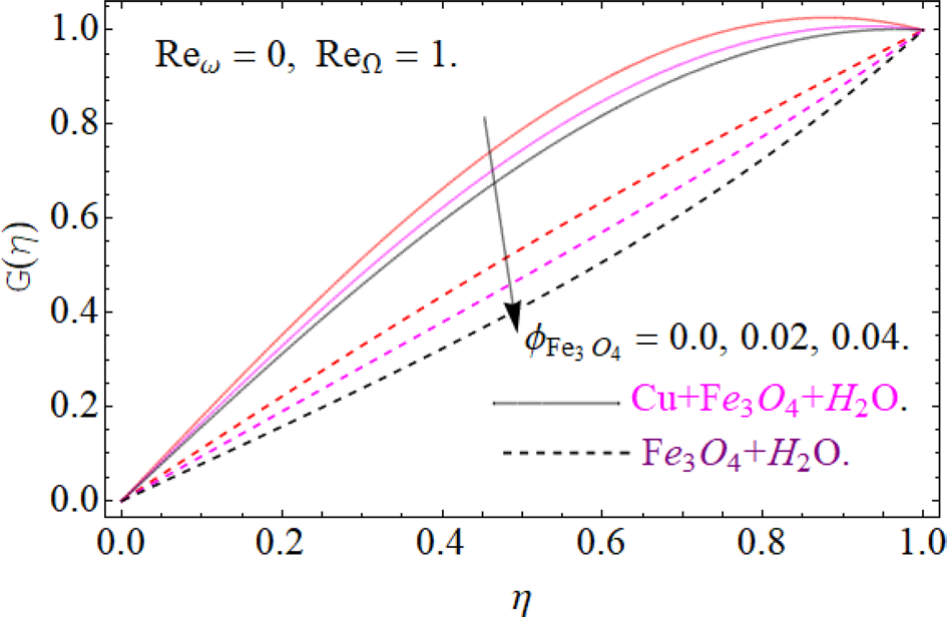
Impression of $\phi_{{Fe_{3} O_{4} }}$ on G(*η*), When $M = 0.2,\,\phi_{Cu} { = 0}{\text{.02,}}\,{\text{Pr = 6}}{.2}$.

**Figure 7:**
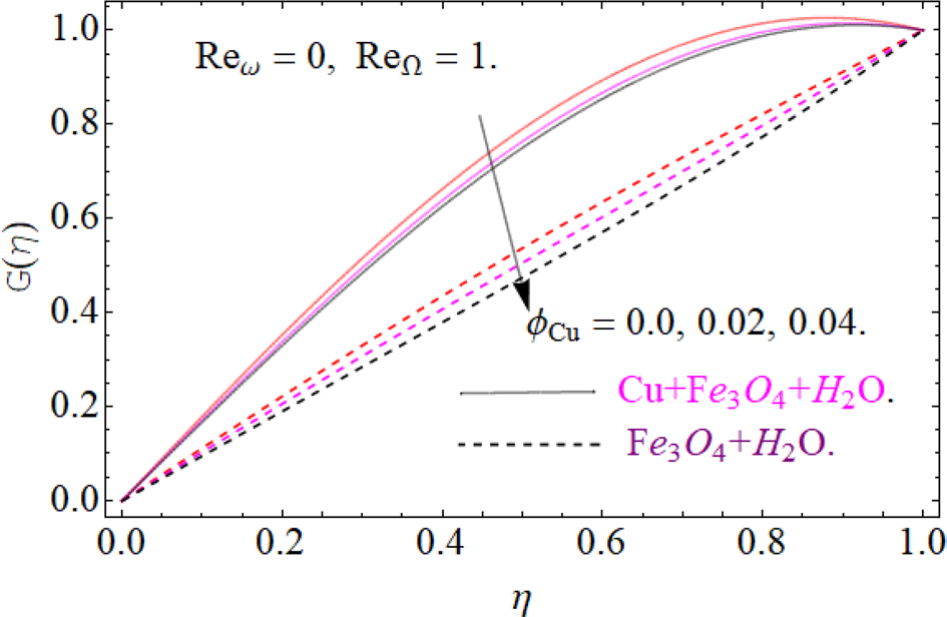
Impression of $\phi_{Cu}$ on G(*η*), When $M = 0.2,\,\phi_{{Fe_{3} O_{4} }} { = 0}{\text{.04,}}\,{\text{Ec = 0}}{.1,}\,{\text{Pr = 6}}{.2}$.

Figures 5, 6 and 7 scrutinized the influence of magnetic parameter M, volume fraction parameter of iron oxide $\phi_{{Fe_{3} O_{4} }}$ and copper $\phi_{Cu}$ on radial velocity $G\left( \eta \right)$ profile respectively. It can be observed that the radial velocity also revealed the same behavior as axial velocity against the nominated parameters.

**Figure 8:**
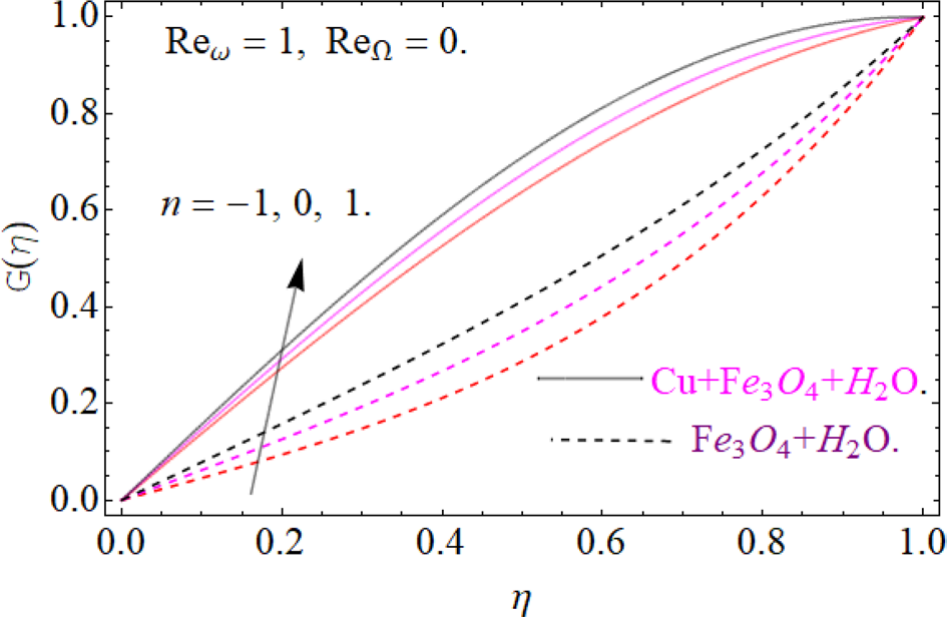
Case (1) Impression over G(*η*), When $M = 0.2,\,\phi_{{Fe_{3} O_{4} }} { = }\phi_{Cu} { = 0}{\text{.02,}}\,{\text{Ec = 0}}{.1,}\,{\text{Pr = 6}}{.2}$.

**Figure 9:**
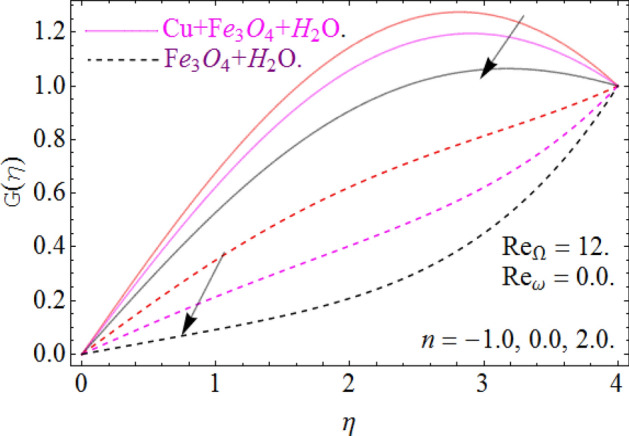
Case (2) on the G(*η*), When $M = 0.2,\,\phi_{{Fe_{3} O_{4} }} { = 0}{\text{.4,}}\phi_{Cu} { = 0}{\text{.2,}}\,{\text{Ec = 0}}{.1,}\,{\text{Pr = 6}}{.2}$.

**Figure 10:**
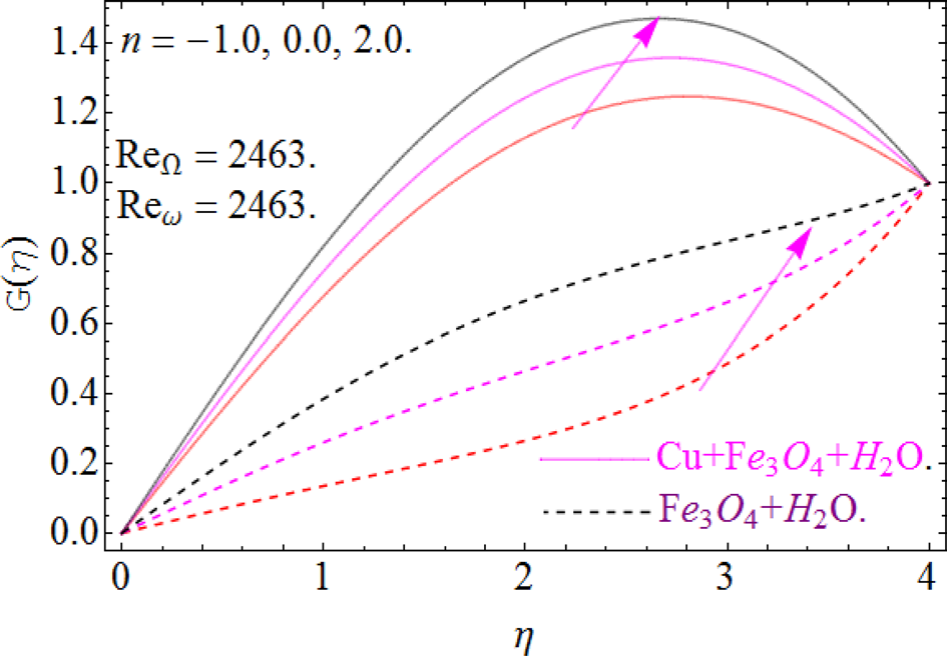
Case (3) on the G(*η*), When $M = 0.2,\,\phi_{{Fe_{3} O_{4} }} { = 0}{\text{.4,}}\phi_{Cu} { = 0}{\text{.2,}}\,{\text{Ec = 0}}{.1,}\,{\text{Pr = 6}}{.2}$.

**Figure 11:**
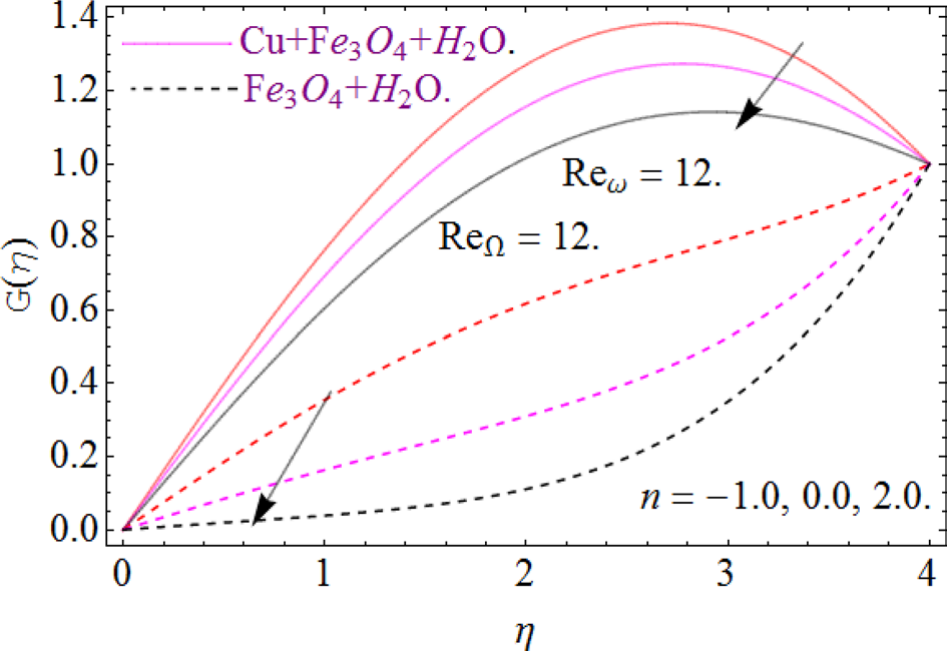
Case (4) on the G(*η*), When $M = 0.2,\,\phi_{{Fe_{3} O_{4} }} { = 0}{\text{.4,}}\phi_{Cu} { = 0}{\text{.2,}}\,{\text{Ec = 0}}{.1,}\,{\text{Pr = 6}}{.2}$.

All four cases regarding to disk, cone angular motion is briefly discussed in Figs. 8, 9, 10 and 11 respectively. Case (1) describes the situation, when the disk is at rest while the cone is rotating. The fluid actually moves between the disk-cone gaps. But the maximum flow intensity is found around the cone, therefore the positive variation in cone velocity ${\text{Re}}_{\Omega } = r^{2} \Omega /\nu$ enhances the radial profile $G\left( \eta \right)$. On the other hand, an opposite trend has been found in case (2), when the cone is at rest, while the disk is in motion with angular velocity ${\text{Re}}_{\omega } = r^{2} \omega /\nu$. According to the no-slip condition, the fluid particles at the cone wall produces some resistance to the flow field. So that’ why such phenomena have been observed. In case (3) both the disk and cone rotate in the same direction, therefore due to the minimum amount of resistance, the flow field illustrates its dominance against ${\text{Re}}_{\Omega } {\text{ and }}{\text{Re}}_{\omega }$ respectively. While in Fig. 11. Case (4) highlights that the counter-rotating of disk and cone effectively reduces the fluid velocity, due the maximum amount of resistance.

**Figure 12:**
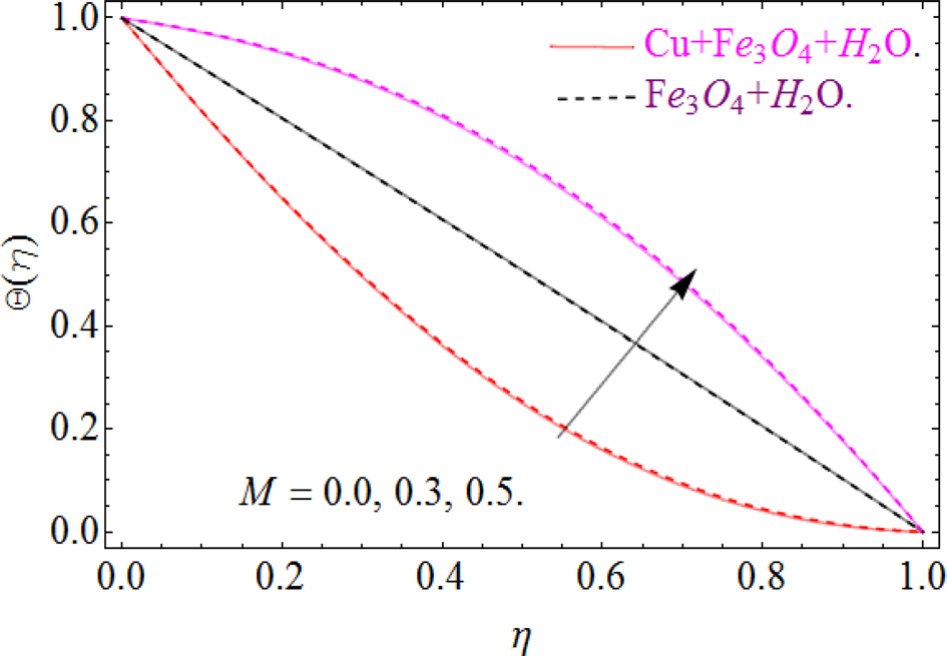
Impression of *M* on Θ(*η*), When $\phi_{{Fe_{3} O_{4} }} = 0.4,\phi_{Cu} = 0.2,\Pr = 6.2,{\text{Re}}_{\omega } = 0.3,{\text{Re}}_{\Omega } = 0.13$.

**Figure 13:**
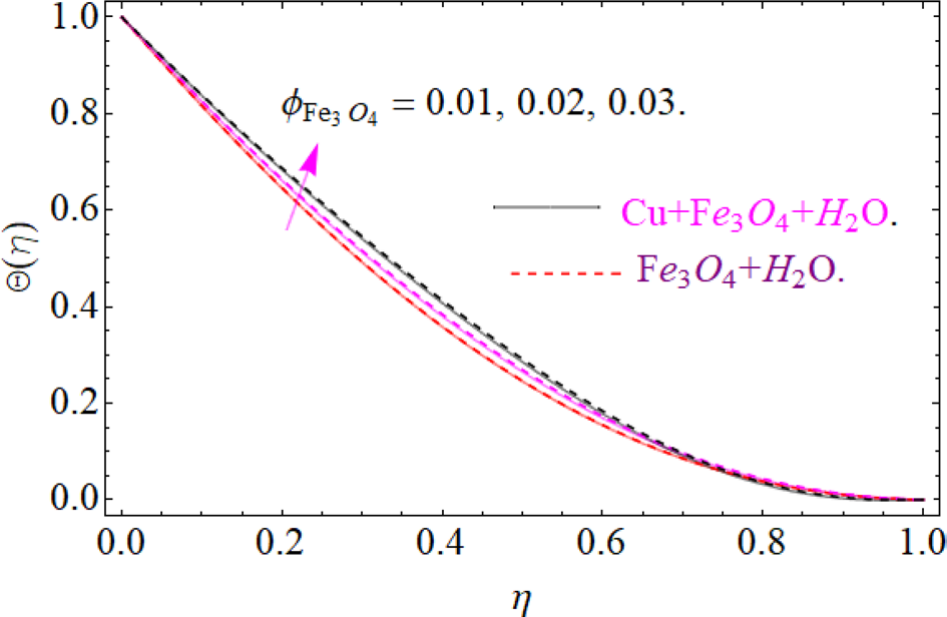
Impression of $\phi_{{Fe_{3} O_{4} }}$ on Θ(*η*), When $M = 0.2,\phi_{Cu} = 0.02,\Pr = 6.2,{\text{Re}}_{\omega } = 0.3,{\text{Re}}_{\Omega } = 0.13$.

**Figure 14:**
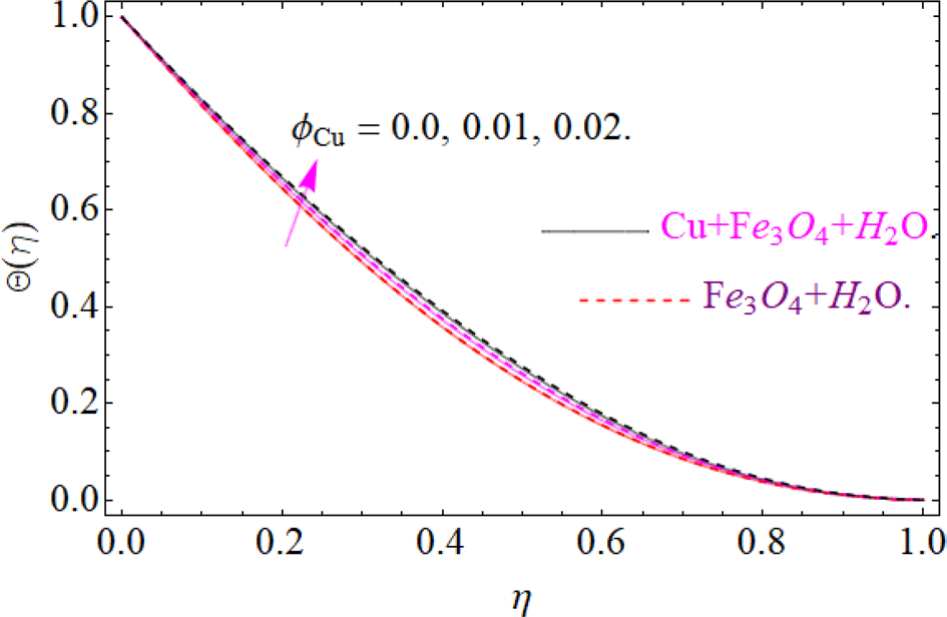
Impression of $\phi_{Cu}$ on Θ(*η*), When $M = 0.2,\phi_{{Fe_{3} O_{4} }} = 0.02,\Pr = 6.2,{\text{Re}}_{\omega } = 0.3,{\text{Re}}_{\Omega } = 0.13$.

**Figure 15:**
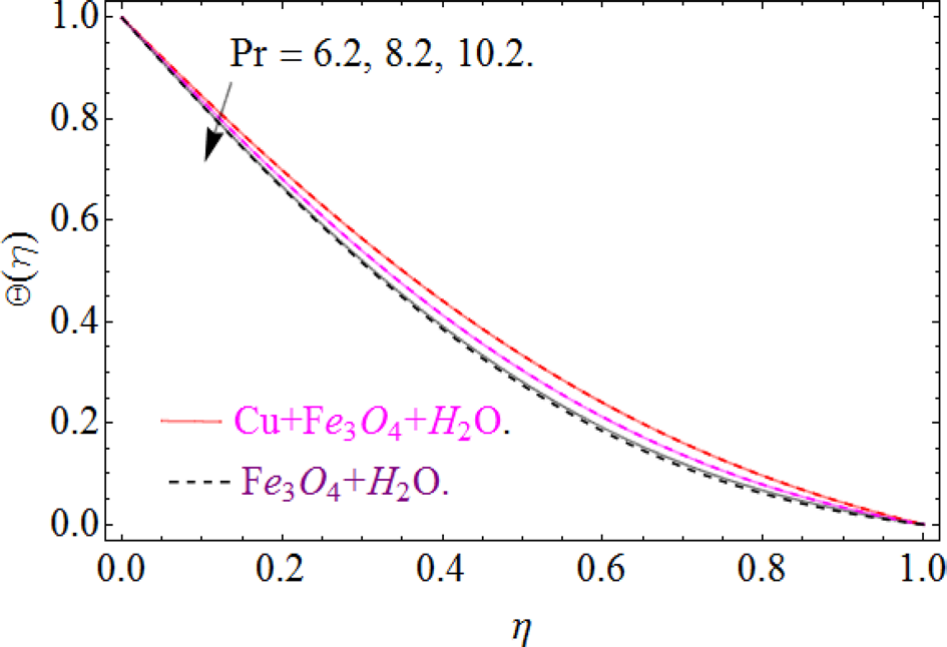
Impression of Pr on Θ(*η*), When $M = 0.2,\phi_{{Fe_{3} O_{4} }} = 0.4,\phi_{Cu} = 0.2,{\text{Re}}_{\omega } = 0.3,{\text{Re}}_{\Omega } = 0.13$.

The nature of temperature distribution $\Theta \left( \eta \right)$ versus magnetic strength M is described via Fig. 12. The Lorentz force retards the fluid to move, as a result, some amount of heat produce, which eventually rises the temperature $\Theta \left( \eta \right)$ of fluid. By adding more quantity of nanoparticles $\left( {\phi_{{Fe_{3} O_{4} }} ,\phi_{Cu} } \right)$ in a carrier fluid enhances the viscosity and heat absorbing capacity of carrier fluid, which raises the fluid temperature elaborated in Figs. 13 and 14 respectively. The dominance of Prandtl number $\Pr$ against temperature distribution is revealed in Fig. 15. Physically, the high Prandtl fluid has always less thermal diffusivity and vice versa.

**Figure 16:**
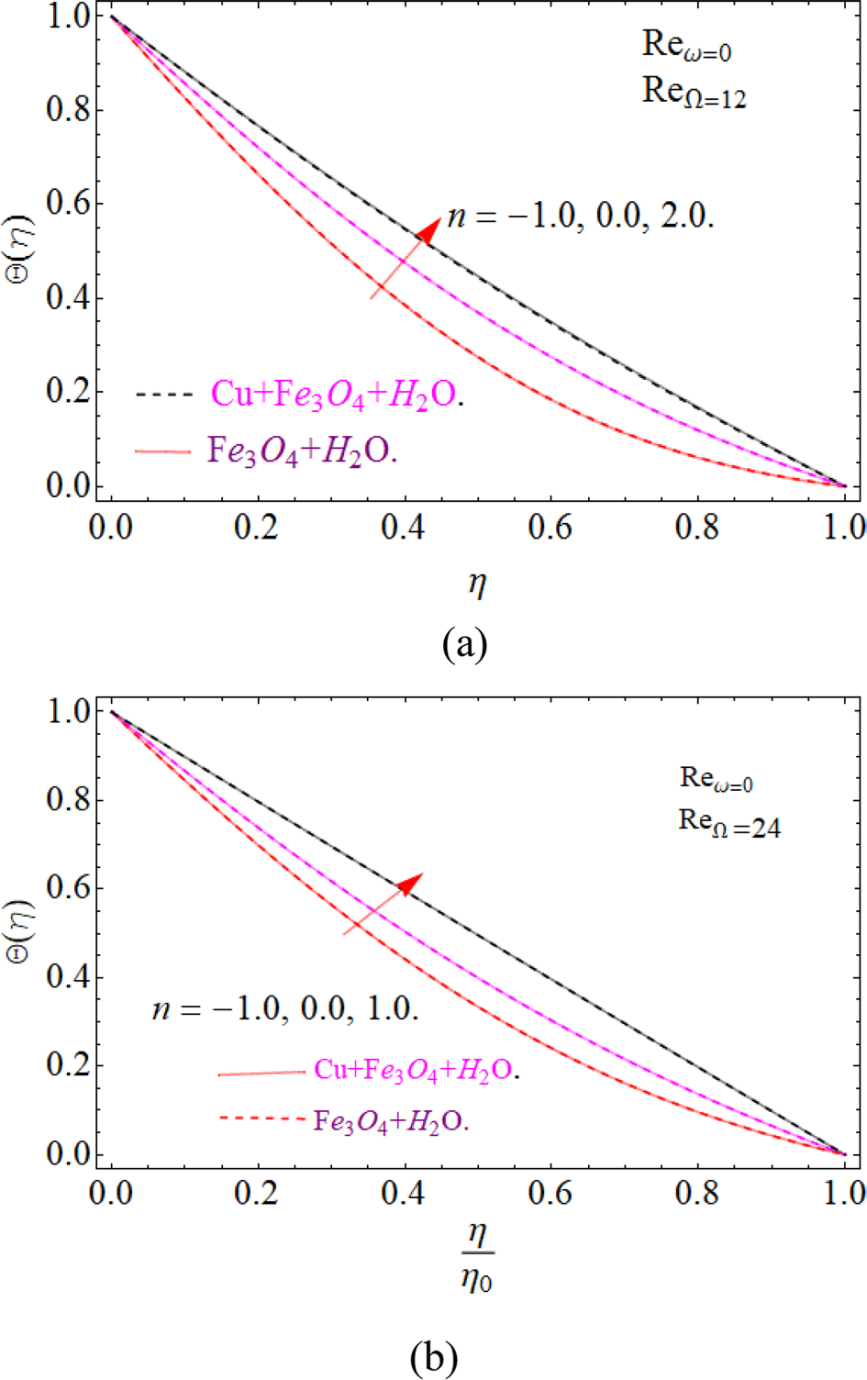
Impression of Θ(*η*) amongst a stationary disk and rotating cone at three different radial exponents. (**a**) Re_Ω_ = 12 and (**b**) Re_Ω_ = 24.

To keep in touch with the published work^45^, we strictly fixed Reynolds number 12 and 2463 throughout the computational work. In case of co-rotation, the ratio of Reynolds number is set up to 1.01, and counter rotating case, it is fixed to -1. We confine the values of power index also to $n = - 1,0,2$, only for comparison purpose with the literature^44,45^. It can be varied with the situation, according to the model. Here case 1 means stationary disk with rotating cone, case 2 stationary cone with rotating disk, case 3 co-rotating disk and cone and case 4. When both disk and cone are counter rotating. Figure 16a,b are sketched, in order to discuss the similarity temperature between a stationary disk and rotating cone (case 1), with varying credit of $\left( {{\text{Re}}_{\omega } } \right)$. In case 1. Temperature field is illustrated within the conical gap from the surface of the cone and disk. It can be seen that the temperature is slightly affected in the increase’s manner throughout the thermal layer in normal apex angles. Although not much effect is perceived in case of minor gap angle. Actually, a critical power index $n = - 1,0,1$, is appearing there, so the heat transmission from the surface of the disk become zero, hence the fluid at the disk surface act as an insulator, because no heat transfer phenomena take place.

**Figure 17:**
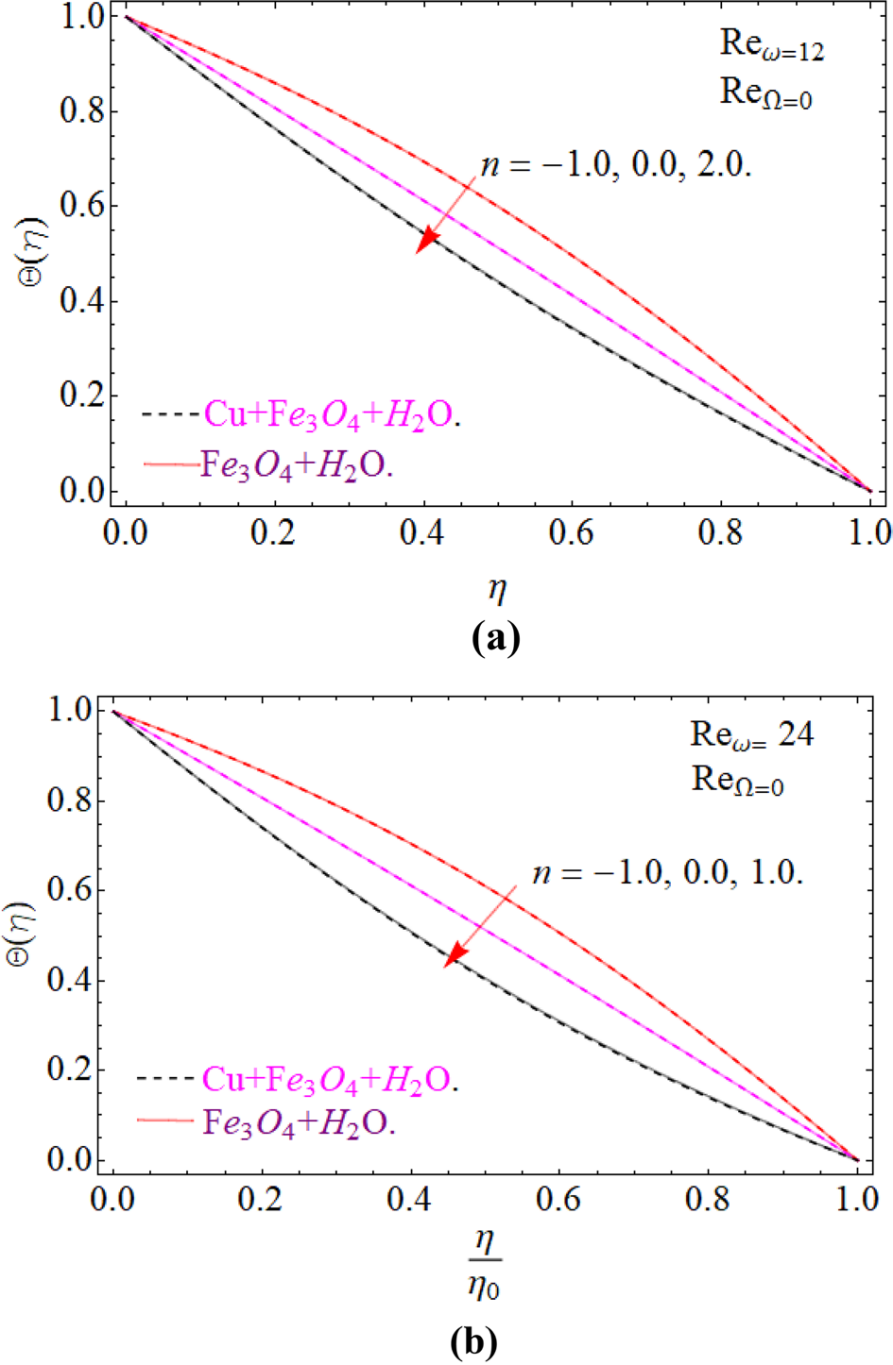
Impression of Θ(*η*) amongst a stationary cone and rotating disk at three different radial exponents. (**a**) Re_Ω_ = 12 and (**b**) Re_Ω_ = 24.

**Figure 18:**
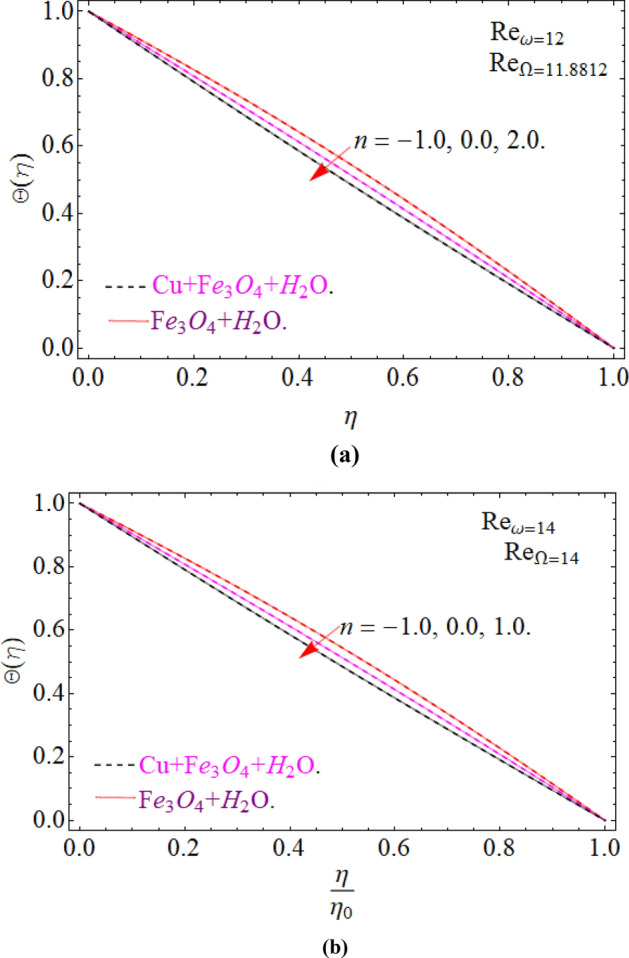
Impression of Θ(*η*) amongst a co-rotating disk and cone at three different radial exponents with Re_*ω*_/Re_Ω_ = 1.01. (**a**) Re_*ω*_ = 12 and (**b**) Re_*ω*_ = 14.

**Figure 19:**
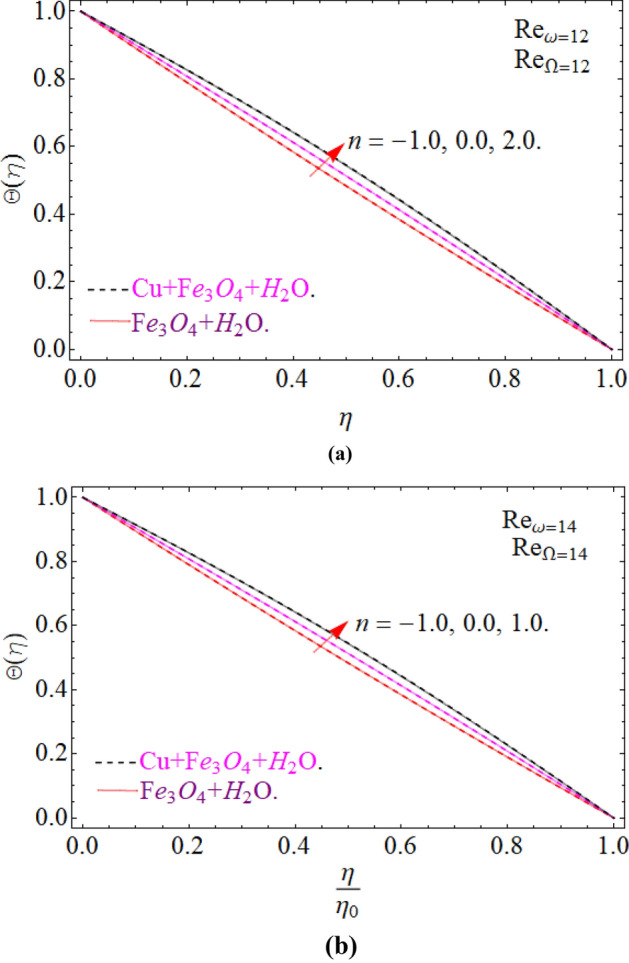
Impression of Θ(*η*) amongst a counter rotating disk and cone at three different radial exponents with Re_*ω*_ = − Re_Ω_. (**a**) Re_*ω*_ = 12 and (**b**) Re_*ω*_ = 14.

**Figure 20:**
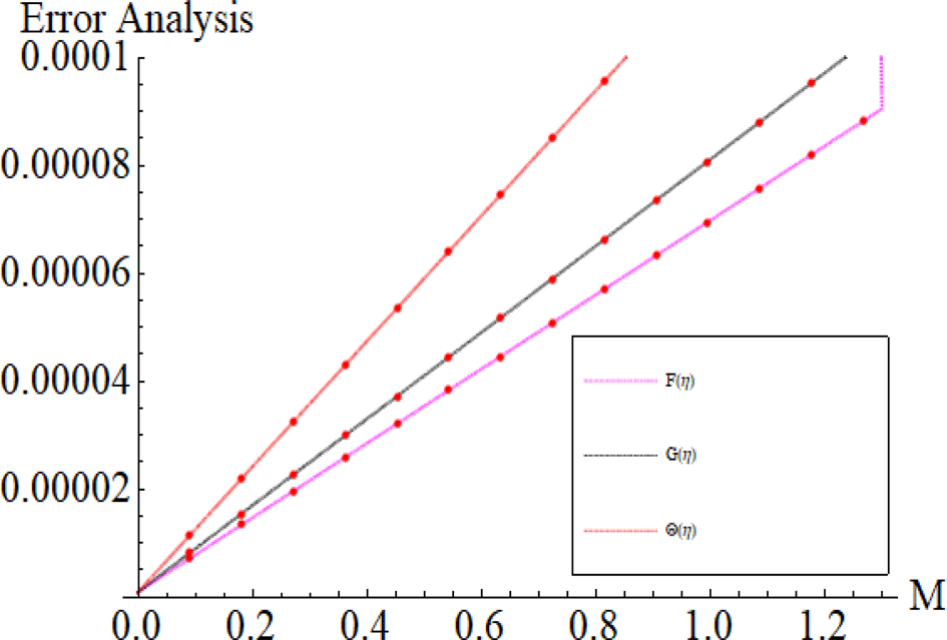
Impression of the parameter *M* range.

**Figure 21:**
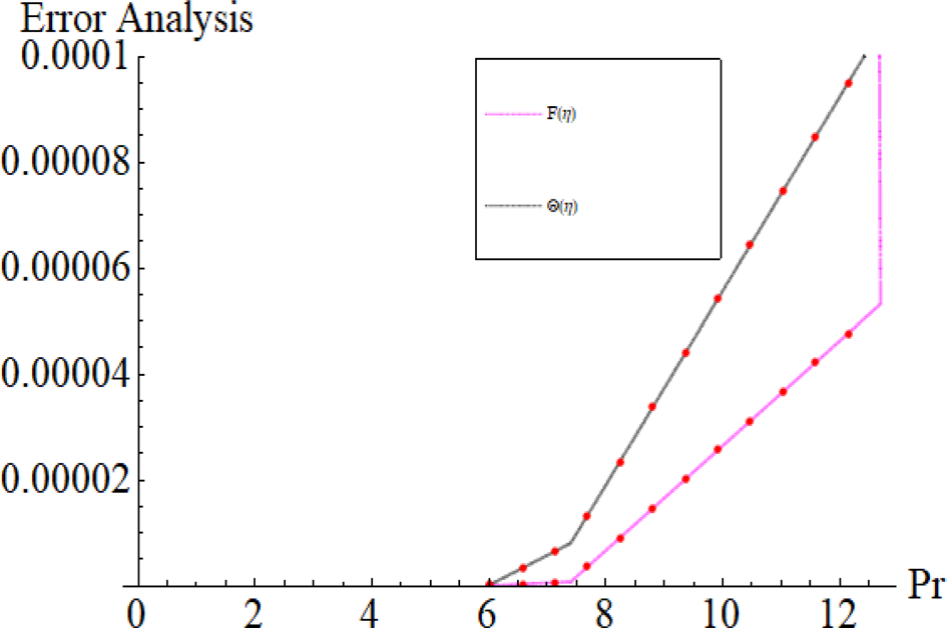
Impression of the parameter Pr range.

**Figure 22:**
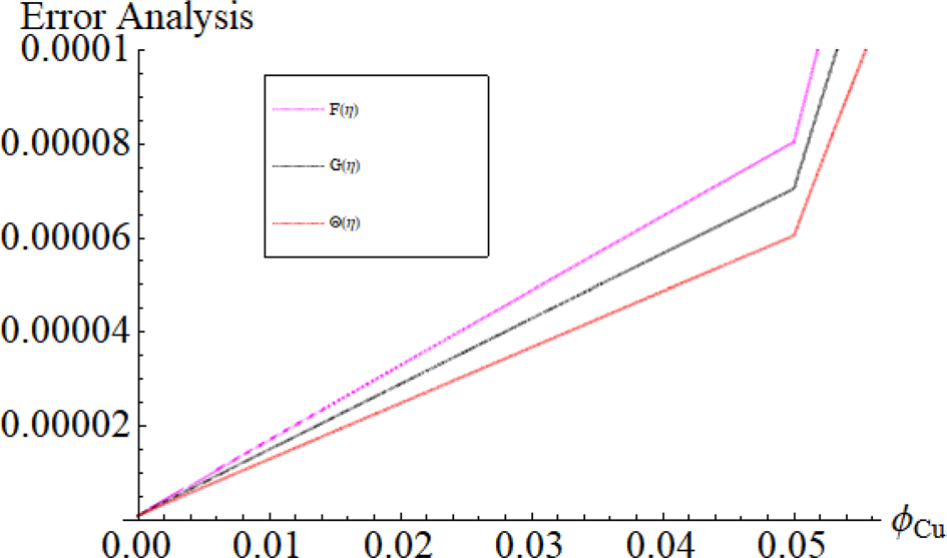
Impression of the parameter *ϕ*_*Cu*_ range.

**Figure 23:**
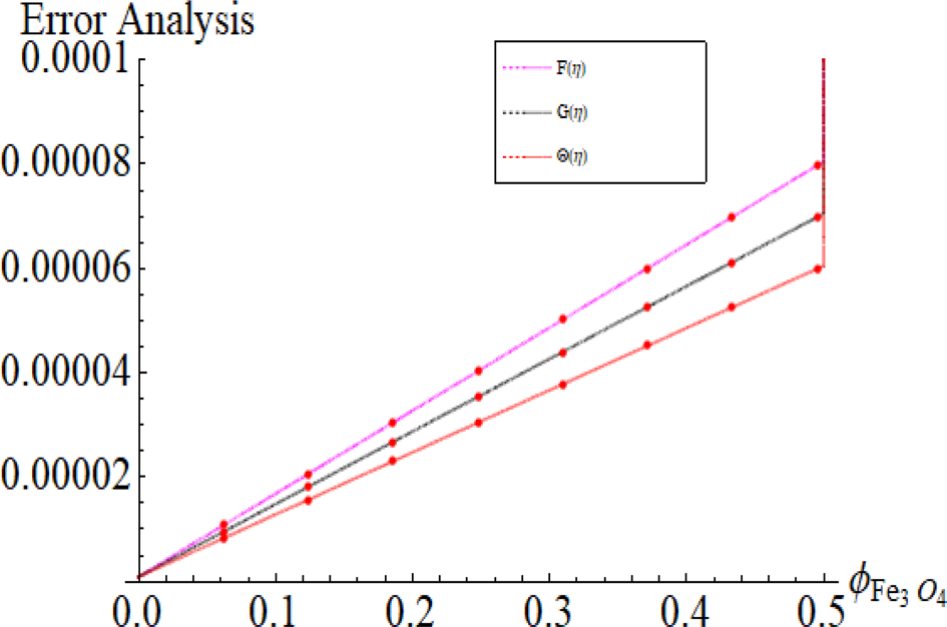
Impression of the parameter $\phi_{{Fe_{3} O_{4} }}$ range.

Figure 17a,b are plotted, in order to scrutinize the temperature profile and heat transfer from the cone and the disk surface within the conical gap, according to case 2 (a stationary cone with the rotating disk). It can be seen that case 2 gains high heat transfer rate only for fixed wall temperature (n = 0). However, cooling process increases for a stationary cone and rotating disk for a high range of radially varying disk temperature distribution. Figure 18 summarized case 3 (co-rotating disk and cone) situation. It is concluded that with the co-rotation of the disk and cone, the temperature of the system rapidly reduces. Finally, the case 4 (both disk and cone are counter rotating) is demonstrated through Fig. 19. When both disk and cone are counter rotating, they produce retardation forces, which opposes the fluid particles to move, as a result the heat produces there, which causes the uprising in temperature. The range of the important physical parameters for the proposed problem is calculated and shown in Figs. 20, 21, 22 and 23. The thermo-physical properties of the base liquid and solid particles are reflected in Table 1. The sum and square of the total residual for the $Fe_{3} O_{4}$ and $Fe_{3} O_{4} + Cu$ are displayed in Tables 2 and 3. These numerical outcomes show the convergence of the HAM method and this convergence, increasing with the increasing number of iterations. Tables 4 and 5 are displayed to show the comparison between the HAM and Numerical (ND-Solve) method for the $Fe_{3} O_{4}$ and $Fe_{3} O_{4} + Cu$ respectively. Again, the absolute error in these tables shows the strong agreement between the HAM and ND-Solve method. Table 6, exhibits the numerical outcomes of the Nusselt number $- \Theta^{\prime } (0)$ at the disc surface for the $Fe_{3} O_{4}$ and $Fe_{3} O_{4} + Cu$ respectively. While Table 7, shows the numerical outcomes of the Nusselt number $- \Theta^{\prime } (1)$ at the cone surface for the $Fe_{3} O_{4}$ and $Fe_{3} O_{4} + Cu$ respectively. The impact of the important physical parameters is calculated and discussed. The larger amount of the Prandtl number declines the heat transfer rate at both the disc and cone surfaces. The increasing in the Eckert number enhancing the heat transfer rate and it happened due to the viscous dissipation term. The impact of the Prandtl number is more efficient using the hybrid nanofluid $Fe_{3} O_{4} + Cu/H_{2} O$ as shown in the Tables 6 and 7. Similarly, the enrichment in the nanoparticle volume fractions enhancing the heat transfer rate and influential improvement achieved using the hybrid nanofluid $Fe_{3} O_{4} + Cu/H_{2} O$. The present study is compared in the Table 8 with the existing literature and closed agreement obtained.

**Table 1:** The numerical properties of water, $Cu$ and $Fe_{3} O_{4}$^46^.

	$\rho$(kg/m^3^)	$C_{p}$ (j/kg K)	$k$ (W/mk)
Water $H_{2} O$	997.1	4179	0.613
Copper *Cu*	8933	385	400
Iron oxide $Fe_{3} O_{4}$	5180	670	9.7

**Table 2:** Total squares residual errors for $Fe_{3} O_{4}$. When $\phi_{{Fe_{3} O_{4} }} = \phi_{Cu} = 0.02,\Pr = 6.2,Re_{\omega } = 0.3,Re_{\Omega } = 0.13.$

$m$	$\varepsilon_{m}^{F} \,Fe_{3} O_{4}$	$\varepsilon_{m}^{G} \,Fe_{3} O_{4}$	$\varepsilon_{m}^{H} \,Fe_{3} O_{4}$	$\varepsilon_{m}^{\Theta } Fe_{3} O_{4}$
4	$5.16485 \times 10^{ - 3}$	$4.88652 \times 10^{ - 7}$	$5.58668 \times 10^{ - 3}$	$4.25884 \times 10^{ - 3}$
8	$3.54455 \times 10^{ - 3}$	$3.95355 \times 10^{ - 9}$	$5.1578 \times 10^{ - 3}$	$2.3672 \times 10^{ - 3}$
12	$1.49664 \times 10^{ - 4}$	$7.42788 \times 10^{ - 11}$	$4.15877 \times 10^{ - 4}$	$3.55206 \times 10^{ - 4}$
15	$6.22764 \times 10^{ - 5}$	$2.86429 \times 10^{ - 11}$	$3.1787 \times 10^{ - 5}$	$4.2556 \times 10^{ - 4}$

**Table 3:** Total squares residual errors for MWCNTs. $\phi_{{Fe_{3} O_{4} }} = \phi_{Cu} = 0.02,\Pr = 6.2,Re_{\omega } = 0.3,Re_{\Omega } = 0.13.$

$m$	$\varepsilon_{m}^{F} \,Fe_{3} O_{4} + Cu$	$\varepsilon_{m}^{G} \,Fe_{3} O_{4} + Cu$	$\varepsilon_{m}^{H} \,Fe_{3} O_{4} + Cu$	$\varepsilon_{m}^{\Theta } Fe_{3} O_{4} + Cu$
4	$2.1142 \times 10^{ - 4}$	$7.6448 \times 10^{ - 8}$	$7.6266 \times 10^{ - 4}$	$3.7438 \times 10^{ - 4}$
8	$4.5848 \times 10^{ - 4}$	$4.48579 \times 10^{ - 8}$	$5.24438 \times 10^{ - 4}$	$2.59443 \times 10^{ - 4}$
12	$3.2239 \times 10^{ - 6}$	$2.22825 \times 10^{ - 10}$	$4.14419 \times 10^{ - 5}$	$3.71532 \times 10^{ - 5}$
15	$2.69422 \times 10^{ - 5}$	$3.41569 \times 10^{ - 11}$	$2.34470 \times 10^{ - 8}$	$3.24407 \times 10^{ - 6}$

**Table 4:** HAM and Numerical comparison for $Fe_{3} O_{4}$: when $\phi_{{Fe_{3} O_{4} }} = \phi_{Cu} = 0.02,\Pr = 6.2,Re_{\omega } = 0.3,Re_{\Omega } = 0.13.$

No	OHAM	Numerical	Absolute error
1	$1.43 \times 10^{ - 10}$	$6.335 \times 10^{ - 16}$	$8.3298 \times 10^{ - 12}$
2	$4.13 \times 10^{ - 11}$	$3.734 \times 10^{ - 19}$	$2.8139 \times 10^{ - 13}$
3	$4.59 \times 10^{ - 11}$	$2.9489 \times 10^{ - 18}$	$4.5682 \times 10^{ - 14}$
4	$7.329 \times 10^{ - 13}$	$8.349 \times 10^{ - 19}$	$7.7659 \times 10^{ - 15}$
5	$6.798 \times 10^{ - 13}$	$4.9378 \times 10^{ - 19}$	$8.8392 \times 10^{ - 15}$
6	$8.23 \times 10^{ - 14}$	$8.1698 \times 10^{ - 20}$	$6.49876 \times 10^{ - 16}$

**Table 5:** HAM and Numerical comparison for $Fe_{3} O_{4} + Cu$: when $\phi_{{Fe_{3} O_{4} }} = \phi_{Cu} = 0.02,\Pr = 6.2,Re_{\omega } = 0.3,Re_{\Omega } = 0.13.$

No	OHAM	Numerical	Error
1	$4.4967 \times 10^{ - 10}$	$5.5748 \times 10^{ - 16}$	$3.88513 \times 10^{ - 12}$
2	$3.4919 \times 10^{ - 11}$	$1.2994 \times 10^{ - 17}$	$7.1943 \times 10^{ - 13}$
3	$7.6945 \times 10^{ - 12}$	$7.6819 \times 10^{ - 18}$	$6.67912 \times 10^{ - 14}$
4	$6.52388 \times 10^{ - 13}$	$3.72981 \times 10^{ - 19}$	$5.96421 \times 10^{ - 15}$
5	$4.9568 \times 10^{ - 14}$	$7.35431 \times 10^{ - 20}$	$4.76187 \times 10^{ - 16}$
6	$8.18435 \times 10^{ - 15}$	$2.8874 \times 10^{ - 21}$	$2.92430 \times 10^{ - 18}$

**Table 6:** Exhibits the Numerical outcomes of Nusselt number $- \Theta^{\prime}\left( 0 \right)$ at the disc surface. When $Re_{\omega } = 0.3,Re_{\Omega } = 0.13.$

$\Pr$	$$\begin{aligned} & - \Theta^{\prime } (0)\,Fe_{3} O_{4} \\ & \phi_{{Fe_{3} O_{4} }} = 0.02 \\ \end{aligned}$$	$$\begin{aligned} & - \Theta^{\prime } (0)\,Fe_{3} O_{4} \\ & \phi_{{Fe_{3} O_{4} }} = 0.03 \\ \end{aligned}$$	$$\begin{aligned} & - \Theta^{\prime } (0)\,Fe_{3} O_{4} + Cu \\ & \phi_{{Fe_{3} O_{4} }} = \phi_{Cu} = 0.02 \\ \end{aligned}$$	$$\begin{aligned} & - \Theta^{\prime } (0)\,Fe_{3} O_{4} + Cu \\ & \phi_{{Fe_{3} O_{4} }} = \phi_{Cu} = 0.03 \\ \end{aligned}$$
6.3	0.966487	0.969312	0.966945	0.971377
6.4	0.965697	0.969587	0.966274	0.969668
6.5	0.964928	0.968862	0.965492	0.959958

**Table 7:** Numerical outcomes of Nusselt number $- \Theta^{\prime}\left( 1 \right)$ at the cone surface. When $Re_{\omega } = 0.3,Re_{\Omega } = 0.13.$

$\Pr$	$$\begin{gathered} - \Theta^{\prime}(1)\,Fe_{3} O_{4} \hfill \\ \phi_{{Fe_{3} O_{4} }} = 0.01 \hfill \\ \end{gathered}$$	$$\begin{gathered} - \Theta^{\prime}(1)\,Fe_{3} O_{4} \hfill \\ \phi_{{Fe_{3} O_{4} }} = 0.02 \hfill \\ \end{gathered}$$	$$\begin{gathered} - \Theta^{\prime}(1)\,Fe_{3} O_{4} + Cu \hfill \\ \phi_{{Fe_{3} O_{4} }} = \phi_{Cu} = 0.01 \hfill \\ \end{gathered}$$	$$\begin{gathered} - \Theta^{\prime}(1)\,Fe_{3} O_{4} + Cu \hfill \\ \phi_{{Fe_{3} O_{4} }} = \phi_{Cu} = 0.02 \hfill \\ \end{gathered}$$
6.3	1.15573	1.25956	2.78864	2.79345
6.4	1.15487	1.24471	2.77196	2.78784
6.5	1.15367	1.23182	2.76845	2.77399

**Table 8:** Comparison of outcomes of Nusselt number at the disc and cone surfaces respectively and those that of outcomes of other studies^47,48^ using only the common parameters.

$n$	$\Theta^{\prime}(0)$^47^	$\Theta^{\prime}(0)$^48^	$\Theta^{\prime}(0)$ Present study	$\Theta^{\prime}(1)$^47^	$\Theta^{\prime}(1)$^48^	$\Theta^{\prime}(1)$ Present study
1	0.757932	0.758845	0.7590312	1.420219	1.421320	1.422431
2	0.766821	0.767734	0.7681201	1.431320	1.432431	1.433542
3	0.775701	0.776623	0.7770100	1.442431	1.443542	1.444653

## Conclusion

In present study real applications are revisited mainly disk-cone apparatus used for the industrial usages. A special type of hybrid nanoliquid containing copper $Cu$ and magnetic ferrite $Fe_{3} O_{4}$ nanoparticles are considered. Which are considered as moving or stationary, in both case counter rotating or co-rotating under the influence of magnetic field. Influences of physical interest variables on the velocity and temperature are highlighted through Figures and Tables. The concluded major findings are as follows:

- The heat transfer rate and velocity of carrier fluid enhanced by an improving quantity of solid nanoparticles $\left( {\phi_{{Fe_{3} O_{4} }} ,\phi_{Cu} } \right)$.
- While the opposite scene is observed in case of magnetic parameter *M*, the positive increment of *M* reduces the velocity of fluid and raises its temperature $\Theta \left( \eta \right)$ due to retardation effect known as Lorentz force.
- The local Reynolds numbers ${\text{Re}}_{\omega } = r^{2} \omega /\nu$ and ${\text{Re}}_{\Omega } = r^{2} \Omega /\nu$ based on the angular velocity of the disk and cone positively influence the radial velocity profile $G\left( \eta \right)$.
- It is concluded that the momentum boundary layer improving with the spinning of the cone and disk in the same direction while the decline in the momentum boundary layer observed in the opposite rotation.
- It can be seen that the temperature is slightly affected in the increases manner throughout the thermal layer in normal tip angles. Although not much impact is perceived in case of minor gap angle, due to the appearance of a critical power index $n = - 1,0,2$. So the heat transfer from the disk surface become zero, hence the fluid at the disk surface act as an insulator, because no heat transferring phenomena take place.
- The suitable range of the physical constraints for the proposed model is calculated which strengthen the convergence of the problem.
